# The value of urinary exosomal microRNA‐21 in the early diagnosis and prognosis of bladder cancer

**DOI:** 10.1002/kjm2.12845

**Published:** 2024-05-27

**Authors:** Fu‐Kan Yang, Chao Tian, Lin‐Xiong Zhou, Tian‐Yu Guan, Gui‐Liu Chen, Yi‐Ying Zheng, Zheng‐Guo Cao

**Affiliations:** ^1^ Department of Urology Guangdong Medical University Zhanjiang China; ^2^ Department of Urology Yuebei people’s hospital Shaoguan China; ^3^ Department of Urology Dongguan People’s Hospital, Guangdong Medical University Dongguan China

**Keywords:** bladder cancer, diagnosis, microRNA‐21, prognosis, urinary exosome

## Abstract

Bladder cancer (BC) poses high morbidity and mortality, with urinary exosomal microRNA (miR)‐21 showing potential value in its diagnosis and prognosis, and we probed its specific role. We prospectively selected 116 BC patients and 116 healthy volunteers as the BC and control groups, respectively. BC urinary exosomal miR‐146a‐5p, miR‐93‐5p, miR‐663b, miR‐21, and miR‐4454 relative expression levels were assessed. The correlations between clinical indexes and urinary exosomal miR‐21, prognostic value of miR‐21, and diagnostic value of the five candidate miRNAs, urine cytology, and miRNA joint diagnostic panel for BC and urinary exosomal miR‐21, miR‐4454, and urine cytology for Ta‐T1 and T2‐T4 stage BC were analyzed. Urinary exosomal miR‐146a‐5p, miR‐93‐5p, miR‐663b, miR‐21, and miR‐4454 were highly expressed in BC patients. miR‐146a‐5p, miR‐93‐5p, miR‐663b, miR‐21, miR‐4454, miRNA combined diagnostic panel, and urine cytology had certain diagnostic value for BC, with miR‐21, miR‐4454, and miRNA co‐diagnostic panel showing the highest diagnostic value. Collectively, urinary exosomal miR‐21 was closely related to Tumor‐Node‐Metastasis staging and grading in BC patients. Urinary exosomal miR‐21 had high diagnostic value for BC and Ta‐T1 and T2‐T4 stage BC, and had high predictive value for BC poor prognosis, providing an effective indicator for the occurrence, development, and prognostic assessment of BC.

## 1 INTRODUCTION

Bladder cancer (BC) ranks among the prevalent genitourinary malignancies, standing as the tenth most common cancer globally, with an estimated 570,000 new cases and 210,000 related deaths annually.^1^ Pathologically, BC encompasses various subtypes, including bladder uroepithelial cell carcinoma, bladder squamous cell carcinoma, and bladder adenocarcinoma, with bladder uroepithelial cell carcinoma accounting for over 90% of cases.^2^ Current diagnostic standards for BC include urine cytology, cystoscopy, and bladder tissue biopsy.^3^ While urine cytology demonstrates high specificity for BC and sensitivity for high‐grade uroepithelial cancer, its sensitivity for low‐grade tumors remains limited. In contrast, cystoscopy and bladder tissue biopsy are invasive procedures requiring technical expertise and incurring high costs, with cystoscopy particularly associated with discomfort, bleeding, and the risk of urinary tract infections, thus constraining its broader application.^4^ The subtle early symptoms of BC often lead to diagnoses at advanced stages, contributing to high recurrence rates and poor prognoses. Consequently, identifying highly sensitive and specific non‐invasive liquid tumor markers for early BC diagnosis and prognosis represents a pressing challenge in clinical practice.

Exploring potential tumor markers for BC in urine presents a promising strategy.^5^ Urinary tumor markers offer advantages such as high reproducibility, ease of sample acquisition, non‐invasiveness, and cost‐effectiveness. As a reservoir for urine, the bladder harbors biochemical markers that may undergo discernible changes upon early carcinogenic transformations. Notably, exosomes, extracellular vesicles ranging from 30 to 150 nm in diameter, are secreted by various cells and abound in body fluids, including the blood and urine of cancer patients. These exosomes are enriched with proteins and nucleic acids that play pivotal roles in cancer progression and metabolic regulation.^6^ The substantial presence of bladder tissue‐derived exosomes in urine may intricately link with BC progression, thereby underscoring the potential of urinary exosomes as biomarkers for BC.^7^ Consequently, urinary exosomes emerge as promising candidates for identifying biomarkers crucial for early BC diagnosis and prognosis.

microRNAs (miRNAs) represent a class of non‐coding single‐stranded RNA molecules, typically around 22 nucleotides in length, encoded by endogenous genes, and implicated in various facets of BC biology.^8^ Exosomal miRNAs derived from BC cells play pivotal roles in promoting tumor growth, metastasis, drug resistance, and immune evasion. Notably, exosomal miR‐21 secreted by BC cells has been shown to activate the PI3K/AKT pathway in macrophages, thereby fostering cancer progression.^9^ In our quest for a reliable urinary exosomal miR serving as a diagnostic and prognostic marker for BC, we conducted a thorough literature review, identifying five miRNAs associated with BC progression: miR‐146a‐5p,^10^ miR‐93‐5p,^11^ miR‐663b,^12^ miR‐21, and miR‐4454.^13^ These miRNAs are known to participate in intercellular communication within tumor microenvironment via the exosomal pathway, prompting speculation about their potential high detection rates in urinary exosomes and their candidacy as diagnostic markers for BC. Furthermore, previous investigations have underscored the significance of miR‐21 as a BC treatment biomarker, owing to its specific oncogene properties implicated in disease development. This miRNA holds promise for noninvasive BC diagnosis and targeted therapy. Nonetheless, studies elucidating the early diagnostic and prognostic value of urinary exosomal miR‐21 in BC remain relatively scarce. Consequently, this study aims to explore the potential value of urinary exosomal miR‐21 in early BC diagnosis and prognosis, aiming to offer novel clinical insights into the quest for early diagnostic and prognostic markers for BC.

## 2 MATERIALS AND METHODS

### 2.1 Ethics statement

All participants provided informed consent after being briefed on the study's objectives. This study was approved by the Academic Ethics Committee of our hospital and conducted in accordance with the principles outlined in the Declaration of Helsinki.

### 2.2 Sample size estimation

Sample size estimation was conducted using a statistical efficiency‐based approach with G*Power 3.0.10 software (University of Düsseldorf, Germany).^14^ The analysis was based on an independent sample *t*‐test, with parameters set as α = 0.05 and 1‐β = 0.95. A medium effect size (*d* = 0.5) was anticipated, with an equal sample size (N1 = N2), with N1 representing the BC group and N2 representing the control group. Considering a 10% attrition rate, the estimated sample size was N1 = N2 = 105. To ensure adequate statistical power, 116 participants were recruited for both the BC and control groups.

### 2.3 Study subjects

A total of 116 BC patients (69 males and 47 females, median age: 58 years, range: 27, 75 years) admitted to our hospital between January 2017 and January 2020 were enrolled in the study as the BC group. Patients were staged according to the 8th edition of the Tumor‐Node‐Metastasis (TNM) criteria and graded based on the 2016 World Health Organization criteria.^15^ Among them, 59 cases were classified as Ta‐T1 stage and 57 cases as T2‐T4 stage, with 48 cases categorized as low‐grade papillary urothelial neoplasia (PUNLMP) and 68 cases as high grade. Additionally, 116 individuals without BC diagnosis or significant abnormalities in physical examination during the same period were included as the control group (84 males and 32 females, median age: 57, range: 26–78 years). There were no significant differences in age or sex ratio between the BC and control groups (Table 1).

**TABLE 1:** Analysis of general and clinical data of subjects in the BC and control groups.

Parameters	Bladder cancer group (*n* = 116)	Control group (*n* = 116)	*p* value
Age (years)	58 (27, 75)	57 (26, 78)	0.963
Male/female	69/47	84/32	0.052
TNM stage, *n* (%)			—
Ta‐T1	59 (50.86)	—	
T2‐T4	57 (49.14)	—	
Pathological grading, *n* (%)			—
PUNLMP, low grade	48 (41.38)	—	
High grade	68 (58.62)	—	

*Note*: The Shapiro–Wilk test was used to test for normal distribution, and normally distributed measurement data were expressed as mean ± standard deviation, and the independent sample *t*‐test was utilized for comparisons between two groups. Enumeration data were expressed as number of cases (*n*) and percentages (%), and comparisons between the two groups were made using the χ^2^ test.

### 2.4 Inclusion and exclusion criteria

Inclusion criteria were as follows: (1) initial diagnosis of BC confirmed by cystoscopy and histopathological examinations; (2) no preoperative chemoradiotherapy, immunotherapy, or other adjuvant treatment; (3) availability of complete case information and follow‐up data. Exclusion criteria were as follows: (1) presence of severe cardiovascular disease, autoimmune disease, diabetes, and urinary tract infection; (2) abnormal liver or kidney function; (3) concurrent presence of other malignant tumors.

### 2.5 Urine cytology examination

Fifteen milliliters of early morning midstream urine samples were collected from all participants and centrifuged at 1500*g* and 4°C for 10 min. The supernatant was discarded, and the precipitate was collected and made into smears within 2 h of sampling. The smears were stained with Wright‐Giemsa Stain Solution (AC11522, Acmec, Wuhan, Hubei, China) and observed under a microscope by two senior pathologists to diagnose suspicious exfoliated cells.

### 2.6 Urine sample collection and exosome extraction

Fifty milliliters of midstream urine samples were collected from each BC patient and control subject in the early morning before surgery. After labeling, the urine was centrifuged at 4°C and 300*g* for 15 min to remove cell debris and impurities, and the supernatant was sub‐packaged and stored at −80°C.

Urinary exosomes were extracted using ultra‐high‐speed differential centrifugation. The frozen urine sample was thawed in a water bath at 37°C and then centrifuged at 4°C and 300*g* for 10 min. The obtained supernatant was further centrifuged at 2000*g* and 4°C for 10 min, followed by centrifugation at 10,000*g* and 4°C for 30 min. The supernatant was then filtered using a 0.22‐μm filter membrane and centrifuged at 4°C and 100,000*g* for 70 min. The precipitate was resuspended with 500 μL of 1 × phosphate‐buffered saline buffer, followed by another centrifugation at 100,000*g* and 4°C for 70 min. Finally, the precipitate was resuspended with 50 μL of RNAase‐free double distilled water and stored at −80°C.

### 2.7 Exosome identification

Urinary exosomes extracted from the −80°C refrigerator were allowed to thaw at room temperature and then diluted 10‐fold. Their morphological structure was observed using transmission electron microscopy (TEM, Leica, Wetzlar, Germany), and their particle size distribution and concentration were assessed by nanoparticle tracking analysis (NTA, NanoSight NS300, Nanosight, Amesbury, UK). Total protein was extracted from exosomes using the Exosomes Protein Extraction Kit (HR8215, Biolab, Beijing, China), and the exosomal protein concentration was measured using bicinchoninic acid kits (P0010, Beyotime, Shanghai, China). The expression levels of exosome surface positive markers CD9 (ab236630, Abcam, Cambridge, UK), CD63 (ab134045, Abcam), and TSG101 (ab125011, Abcam), and the negative marker Calnexin (ab13504, Abcam) were examined by Western blot, with H&L‐coupled IgG (ab6721, Abcam) as a secondary antibody. The identification of urinary exosomes was confirmed using the aforementioned methods.

### 2.8 Reverse transcription quantitative polymerase chain reaction (RT‐qPCR)

The relative expression levels of miR‐146a‐5p, miR‐93‐5p, miR‐663b, miR‐21, and miR‐4454 in urinary exosomes were measured by RT‐qPCR. Urinary exosomes stored at −80°C were dissolved in a water bath at 37°C, and total RNA was extracted using the Total RNA Extraction Kit (abs60263‐100 T, Absin Bioscience, Shanghai, China), with its concentration determined using an ultra‐micro spectrophotometer (NanDrop one, Thermo Scientific, Waltham, MA, USA). cDNA was synthesized by reverse transcription using the miRNA RT‐qPCR kit (RiboBio, Guangzhou, Guangdong, China). Then, 2 μL of fivefold diluted cDNA was added to a qPCR reaction system consisting of 12.5 μL of SYBR Premix Ex Taq II, 0.5 μL of Dye II, 2 μL of 5 μM forward primer, and 1 μL of 10 μM Uni‐miR RT‐qPCR Primer. The reaction conditions included 40 cycles of 95°C, 30 s, 95°C, 5 s, and 57°C for 34 s under cycling conditions of 7 μL ddH_2_O.^16^ The relative expression patterns of target miRNAs were calculated using the 2^−ΔCT^ method,^17^, ^18^ with U6 used as the internal reference gene for urinary exosomal miRNAs. To verify the stability of U6 as an exosomal reference in urine, 20 BC patients and 20 control subjects were randomly selected, with cel‐miR‐39‐3p regarded as an exogenous internal reference for verification.^19^ After extraction, RNA was added with 1 μL of external reference cel‐miR‐39‐3p (XY‐RDM0000C, BioVendor, Czech Republic), and subjected to reverse transcription to synthesize cDNA. Then, 2 μL of cDNA, diluted five times, was added to the qPCR reaction system for subsequent cycling. The results were analyzed using the 2^−ΔCT^ method. The results indicated no significant difference in the relative expression of miRNA‐21 after using the exogenous internal reference cel‐miR‐39‐3p compared to using U6 as the internal reference (*p* > 0.05, NS for no significant difference), confirming the good stability of U6 as an internal reference for urinary exosomes (Figure S1). The primers were synthesized by RiboBio, and the specific primer information is provided in Table 2.

**TABLE 2:** Primer sequences.

Gene	Forward 5′–3′	Reverse 5′–3′
*miR‐146a‐5p*	GAGAACTGAATTCCATGG	GAACATGTCTGCGTATCTC
*miR‐93‐5p*	CGCAAAGTGCTGTTCGTGC	AGTGCAGGGTCCGAGGTATT
*miR‐663b*	GGTGGCCCGGCCGTGC	TATCCTTGTTGACGACTCCTTGAC
*miRNA‐21*	ACACTCCAGCTGGGTAGCTTATCAGACTGA	TGGTGTCGTGGAGTCG
*miR‐4454*	GATCCGAGTCACGGCACCAA	CAGTGCAGGGTCCGAGGTATTC
*U6*	CTCGCTTCGGCAGCACA	AACGCTTC ACGAATTTGCGT
*cel‐miR‐39‐3p*	GTCGTATCCAGTGCAGGGTCCG	UCACCGGGUGUAAAUCAGCUUG

### 2.9 Definition of follow‐up and adverse outcomes

All included BC patients were followed up for 3 years postoperatively, spanning from February 2017 to February 2023. Cystoscopy was performed 1 month postoperatively to determine the presence of recurrence and symptoms, followed by home reviews every 3 months and monthly telephone follow‐ups. In the 2nd to 3rd years, patients were reviewed at home every 6 months and followed up by telephone every 3 months. The follow‐up endpoint was patient death, and patient recurrence or death after surgery was defined as an adverse outcome.

### 2.10 Statistical analysis

Data were statistically analyzed and plotted using SPSS 22.0 statistical software (IBM Corp. Armonk, NY, USA), GraphPad Prism 8.0 software (GraphPad Software Inc., San Diego, CA, USA), and MedCalc software (22.2, MedCalc software Ltd, Ostend, Belgium). The Shapiro–Wilk test was used to test for normal distribution, with normally distributed measurement data expressed as mean ± standard deviation, and compared between two groups using the independent sample *t*‐test. Enumeration data were expressed as the number of cases (*n*) and percentages (%), with comparisons between two groups conducted using the chi‐square test. *p* was a two‐sided test, and *p* < 0.05 was considered statistically significant.

The diagnostic value of urinary exosomal miRNAs and urine cytology for BC and its staging was evaluated using receiver operating characteristic (ROC) curves. Logit regression analysis was employed to establish a regression model panel to analyze its ROC diagnostic value for BC. The area under the curve (AUC) variability was analyzed using Medcalc software (Solvusoft, Los Angeles, CA, USA).

The risk factors for postoperative recurrence in BC patients were analyzed using univariate and multivariate Cox regression models. BC patients were divided into low‐miR‐21 and high‐miR‐21 groups using the median urinary exosomal miR‐21 as the dividing line. Kaplan–Meier survival curves were implemented to analyze the differences in the 3‐year overall survival rates of BC patients between the two groups.

## 3 RESULTS

### 3.1 Identification of urinary exosomes

**FIGURE 1:**
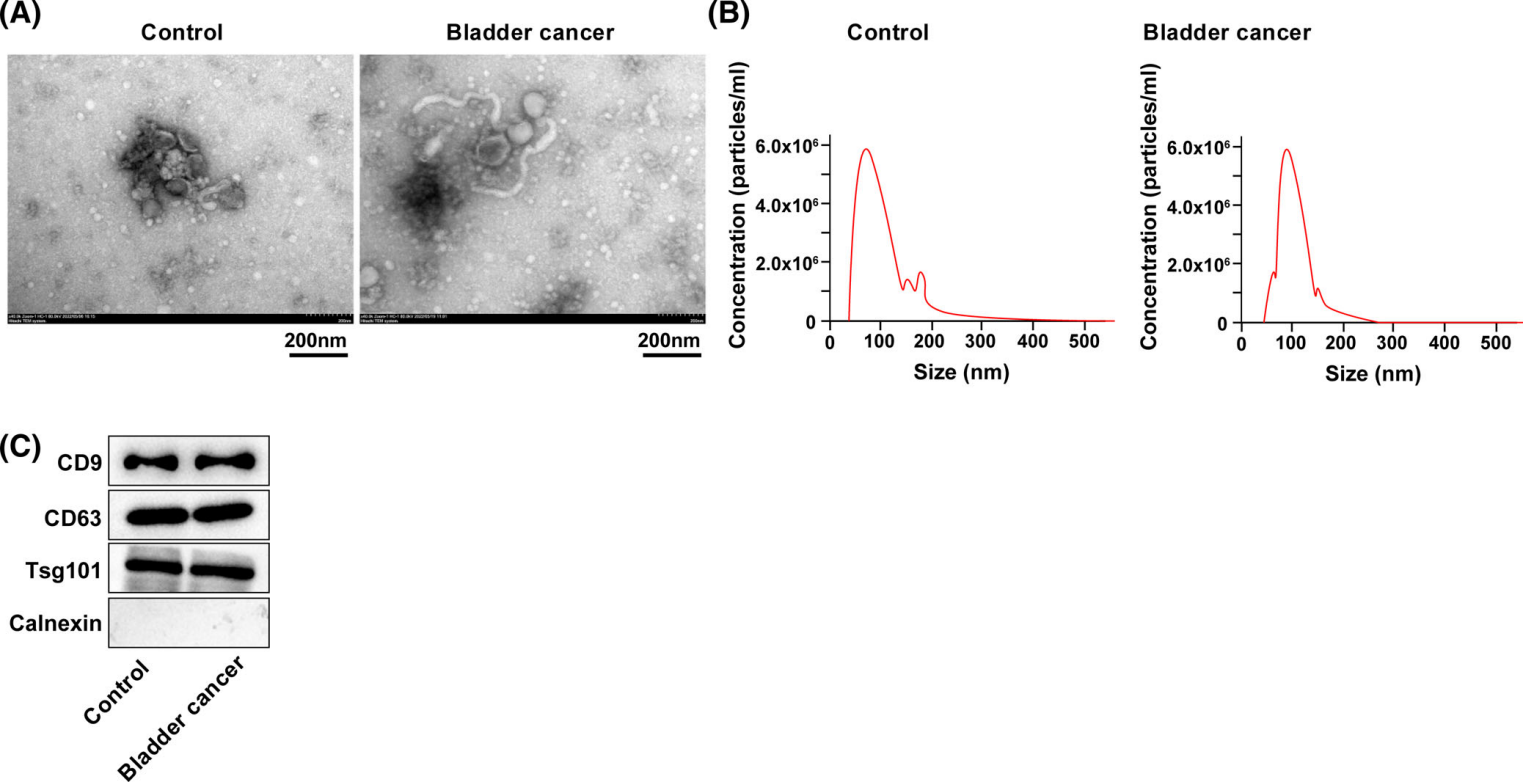
Identification of urinary exosomes. (A) Morphological structure of exosomes observed using transmission electron microscopy (TEM); (B) particle size distribution of exosomes analyzed by nanoparticle tracking analysis (NTA); (C) protein expression levels of exosome positive markers CD9, CD63, TSG101, and negative marker Calnexin tested by Western blot.

Urinary exosomes were successfully extracted from urine samples of 116 BC patients and 116 healthy control subjects using ultra‐high‐speed differential centrifugation. TEM analysis revealed a vesicular bilayer membrane structure with typical exosomal morphological features (Figure 1A). NTA results showed that the main particle size distribution of the extracted exosomes in the control and BC groups ranged from 30 to 200 nm and 25 to 200 nm, respectively (Figure 1B). Western blot analysis confirmed positive expression of exosome surface markers CD9, CD63, and TSG101, along with negative expression of the negative marker Calnexin (Figure 1C), validating the successful extraction of urinary exosomes.

### 3.2 Various miRNAs upregulated in urinary exosomes of BC patients

**FIGURE 2:**
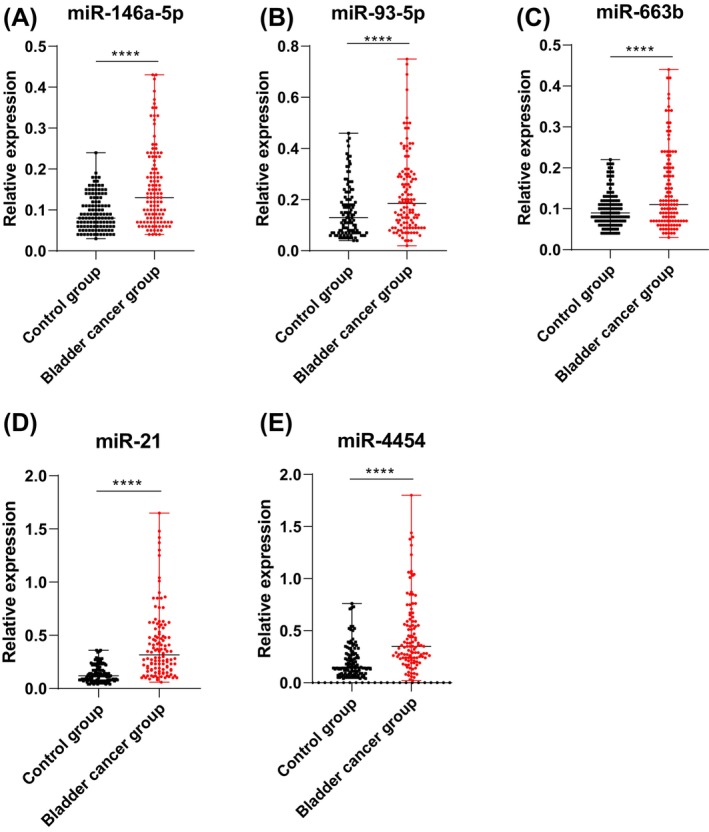
Upregulation of various miRNAs in urinary exosomes of BC patients. (A–E) Relative expression levels of miR‐146a‐5p (A), miR‐93‐5p (B), miR‐663b (C), miR‐21 (D), and miR‐4454 (E) in exosomes assessed by reverse transcription quantitative polymerase chain reaction (RT‐qPCR). N = 116. Non‐normally distributed measurement data represented by quartile (median value [minimum value, maximum value]). Comparisons between groups made using the Mann–Whitney *U* test. ****p < 0.0001.

Several miNRAs closely associated with BC progression, including miR‐146a‐5p,^10^ miR‐93‐5p,^11^ miR‐663b,^12^ miR‐21, and miR‐4454,^13^ were selected as candidate miRNAs for BC diagnosis due to their reported high expression in BC tissues and involvement in intercellular communication via the exosomal pathway in the tumor microenvironment. RT‐qPCR analysis revealed significantly higher relative expression levels of miR‐146a‐5p (0.13 [0.04, 0.43]), miR‐93‐5p (0.19 [0.02, 0.75]), miR‐663b (0.11 [0.03, 0.44]), miR‐21 (0.32 [0.06, 1.65]), and miR‐4454 (0.35 [0.02, 1.80]) in urinary exosomes of the BC group compared to miR‐146a‐5p (0.08 [0.03, 0.24]), miR‐93‐5p (0.13 [0.04, 0.46]), miR‐663b (0.09 [0.04, 0.22]), miR‐21 (0.12 [0.04, 0.36]), and miR‐4454 (0.15 [0.04, 0.76]) in the control group (Figure 2A–E, all *p* < 0.0001). Notably, miR‐21 and miR‐4454 exhibited the largest differences in relative expression changes between the two groups.

### 3.3 Urinary exosomal miR‐21, miR‐4454, and exosome miRNA diagnostic panel (Emdp‐miR) exhibit high diagnostic value for BC

**FIGURE 3:**
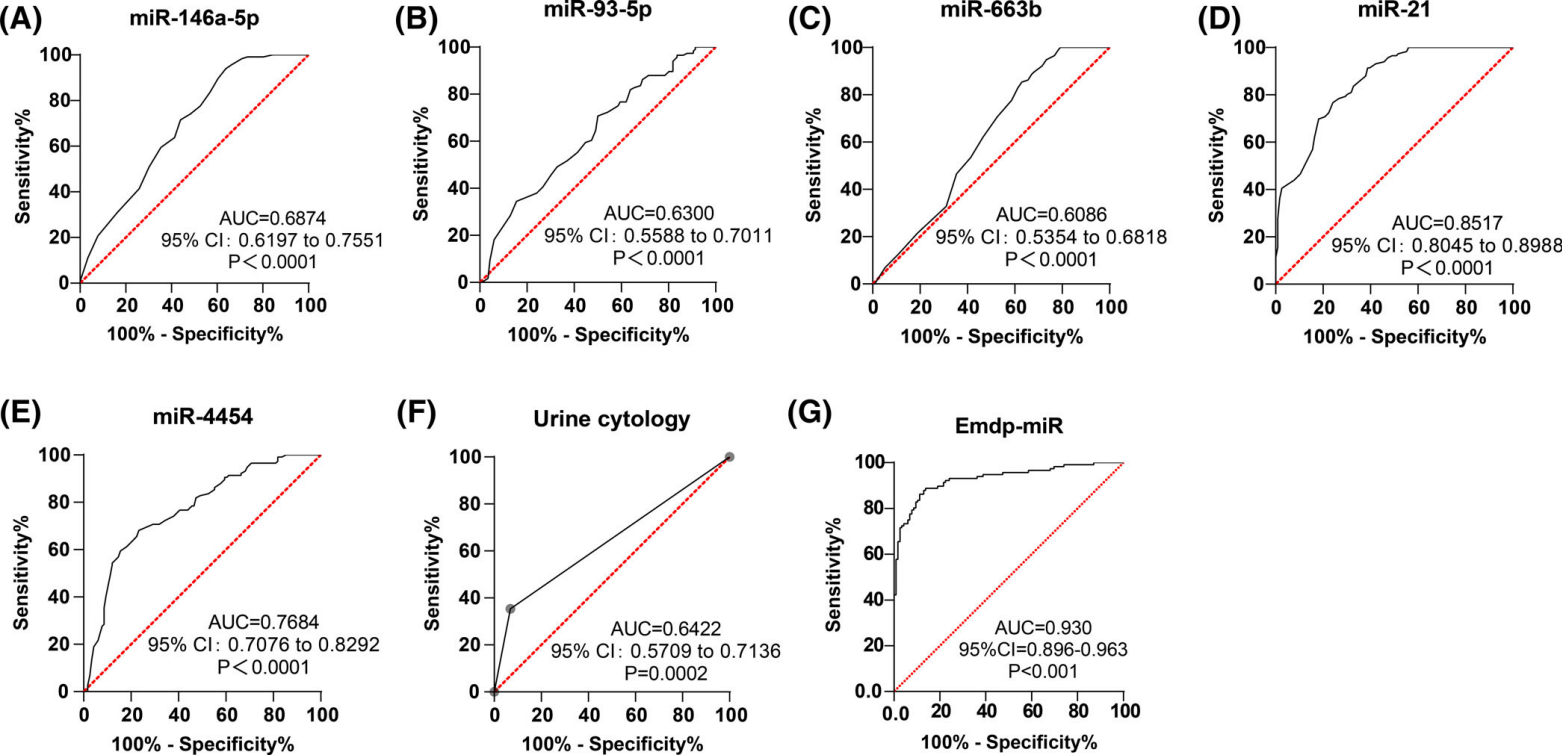
High diagnostic value of urinary exosomal miR‐21, miR‐4454, and Emdp‐miR for BC. (A–G) Receiver operating characteristic (ROC) curve analysis of miR‐146a‐5p (A), miR‐93‐5p (B), miR‐663b (C), miR‐21 (D), miR‐4454 (E), urine cytology (F), and Emdp‐miR (G) for BC diagnostic value.

We assessed the diagnostic value of miR‐146a‐5p, miR‐93‐5p, miR‐663b, miR‐21, miR‐4454, and urine cytology for BC using ROC curves. The AUC of miR‐146a‐5p for BC diagnosis was 0.6874, with a sensitivity of 36.21%, a specificity of 93.97%, and a cut‐off value of 0.16 (Figure 3A, *p* < 0.0001); the AUC of miR‐93‐5p for BC diagnosis was 0.6300, with a sensitivity of 50.00%, a specificity of 70.69%, and a cut‐off value of 0.18 (Figure 3B, *p* < 0.0001); the AUC of miR‐663b for BC diagnosis was 0.6086, with a sensitivity of 37.07%, a specificity of 85.34%, and a cut‐off value of 0.14 (Figure 3C, *p* < 0.0001); the AUC of miR‐21 for BC diagnosis was 0.8517, with a sensitivity of 75.86%, a specificity of 76.72%, and a cut‐off value 0.17 (Figure 3D, *p* < 0.0001); the AUC of miR‐4454 for BC diagnosis was 0.7684, with a sensitivity of 76.72%, a specificity of 68.10%, and a cut‐off value 0.23 (Figure 3E, *p* < 0.0001); the AUC of urine cytology for BC diagnosis was 0.6422, with a sensitivity of 35.34% and a specificity of 93.10% (Figure 3F, *p* = 0.0002). To further develop a more suitable diagnostic model, we used miR‐146a‐5p, miR‐93‐5p, miR‐663b, miR‐21, and miR‐4454 for regression analysis to establish a regression model for the diagnosis and prediction of BC. However, the correlation of miR‐663b is limited, so we used miR‐146a‐5p, miR‐93‐5p, miR‐21, and miR‐4454 to establish a regression model panel. The equation was Logit (*P* = BC) = 14.133 × miR‐146a‐5p (2^−ΔCT^) + 3.697 × miR‐93 (2^−ΔCT^) + 11.924 × miR‐21 (2^−ΔCT^) + 3.961 × miR‐4454 (2^−ΔCT^) − 6.119, which was named Emdp‐miR. The AUC of Emdp‐miR for the diagnosis of BC was 0.930, with a sensitivity of 88.79% and a specificity of 86.21% (Figure 3G, *p* < 0.0001). Comparative analysis (Table 3) revealed that Emdp‐miR outperformed miR‐21, miR‐4454, and urine cytology in BC diagnosis (all *p* < 0.001).

**TABLE 3:** ROC curve analysis of AUC of miR‐21, miR‐4454, Emdp‐miR, and urine cytology for the diagnosis of BC by Medcalc software.

Pairwise comparison of ROC curves	*p* value
miR‐21 versus urine cytology	<0.0001
miR‐4454 versus urine cytology	0.0021
miR‐21 versus miR‐4454	0.0274
Emdp‐miR	<0.0001
miR‐21 versus Emdp‐miR	0.0006
miR‐4454 versus Emdp‐miR	<0.0001
Urine cytology versus Emdp‐miR	<0.0001

### 3.4 Urinary exosomal miR‐21 demonstrates high diagnostic value for early‐stage and late‐stage BC

**FIGURE 4:**
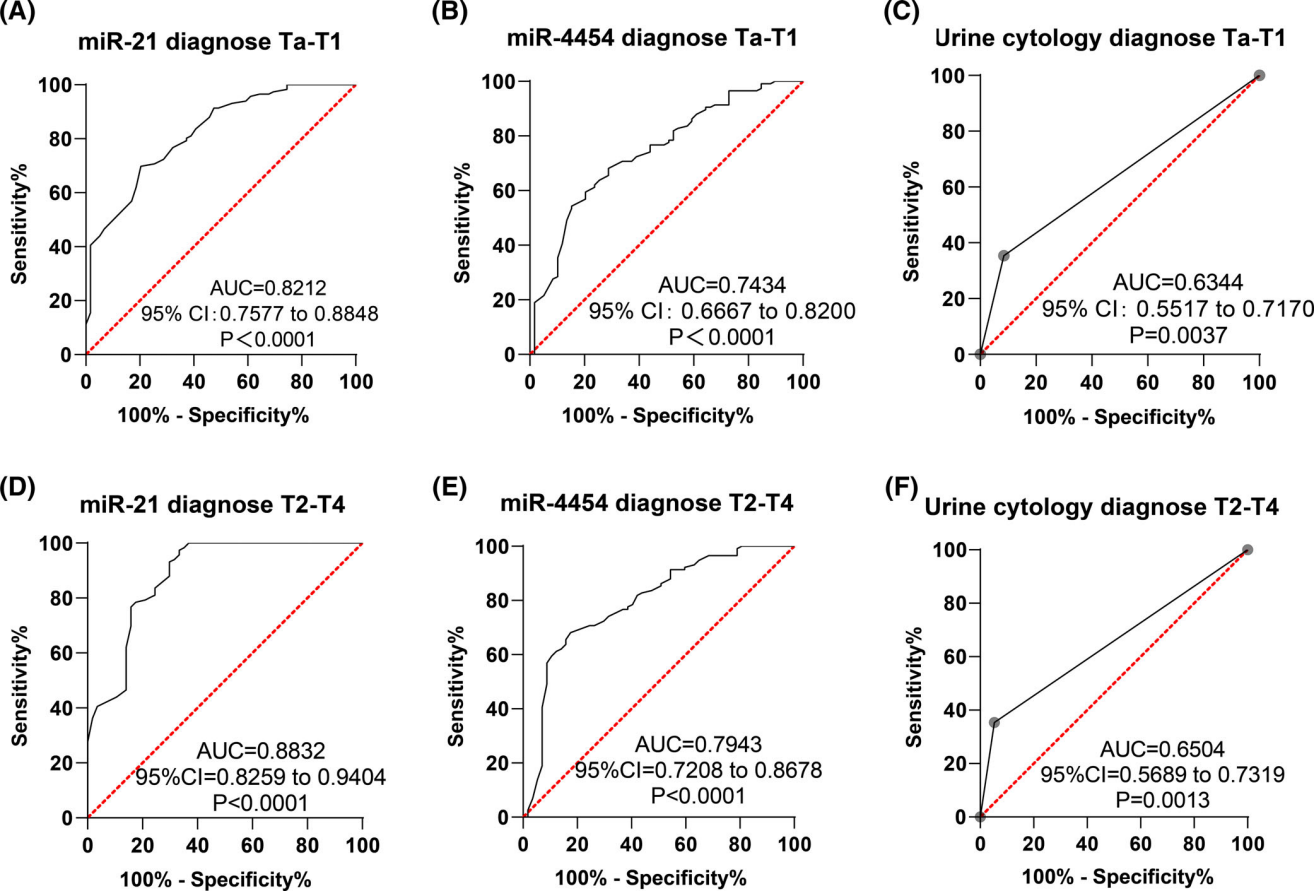
Diagnostic value of urinary exosomal miR‐21, miR‐4454 and urine cytology for BC at Ta‐T1 and T2‐T4 stage by ROC curve analysis.

Analysis of miR‐21, miR‐4454, and urine cytology for BC at Ta‐T1 stage and T2‐T4 stage showed AUCs of 0.8212 and 0.8832 for miR‐21, 0.7434 and 0.7943 for miR‐4454, and 0.6344 and 0.6504 for urine cytology, respectively. The sensitivity, specificity, and cut‐off values varied for each marker across stages (Figure 4A–F). Comparative analysis revealed significantly higher AUCs for urinary exosomal miR‐21 and miR‐4454 compared to urine cytology (*p* < 0.05). While no significant difference was observed between miR‐21 and miR‐4454 (*p* = 0.1069, 0.0587) (Table 4), miR‐4454 exhibited lower specificity. Thus, urinary exosomal miR‐21 showed high diagnostic value for both Ta‐T1 stage and T2‐T4 stage BC.

**TABLE 4:** Analysis of ROC curve of miR‐21, miR‐4454 and urine cytology for diagnosis of Ta‐T1 and T2‐T4 stage BC by Medcalc software.

Pairwise comparison of ROC curves	Ta‐T1 stage *p* value	T2‐T4 stage *p* value
miR‐21 versus urine cytology	<0.0001	<0.0001
miR‐4454 versus urine cytology	0.0267	0.0022
miR‐21 versus miR‐4454	0.1069	0.0587

### 3.5 Association of urinary exosomal miR‐21 with clinical profiles of BC patients

**FIGURE 5:**
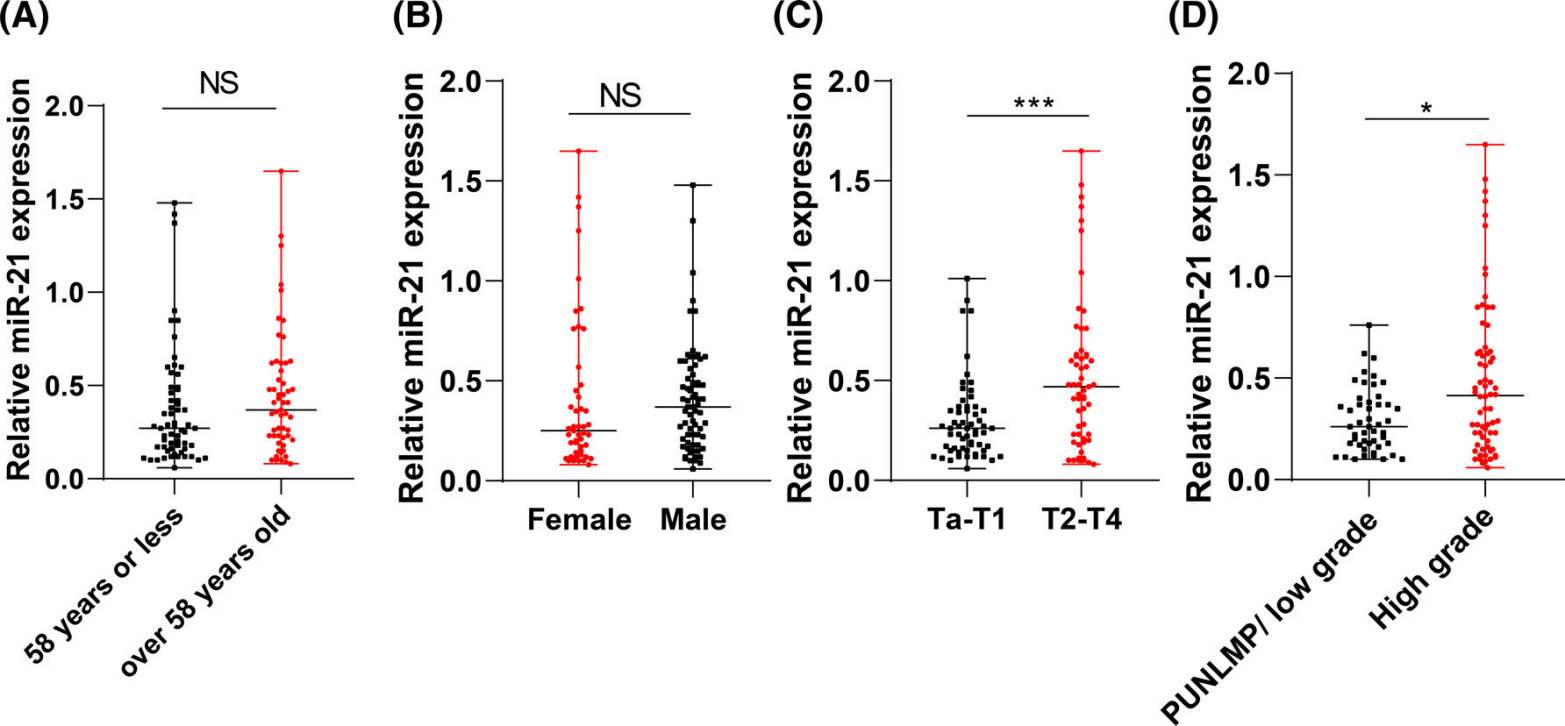
Association between urinary exosomal miR‐21 level and TNM staging and pathological grading of BC. (A) Relative expression of urinary exosomal miR‐21 in BC patients of different age groups assessed by RT‐qPCR, with 58 years as median age; (B) relative expression of urinary exosomal miR‐21 in male and female BC patients tested by RT‐qPCR; (C) relative expression of urinary exosomal miR‐21 in BC patients at Ta‐T1 stage or T2‐T4 stage determined by RT‐qPCR; (D) relative expression of urinary exosomal miR‐21 in BC patients with PUNLMP/low grade or high grade measured by RT‐qPCR. Measurement data of non‐normal distribution expressed by quartile (median value [minimum, maximum]). Comparisons between groups were performed using the Mann–Whitney *U* test. NS, no significant difference; **p < 0.001, *p < 0.05.

We investigated the correlation between age, sex, TNM stage, pathological grading, and urinary exosomal miR‐21 in BC patients. Results showed no significant difference in miR‐21 expression between age groups or genders. However, elevated miR‐21 expression was associated with advanced TNM stage and high‐grade BC (Figure 5A–D, *p* > 0.05). Thus, urinary exosomal miR‐21 levels correlated with TNM staging and pathological grading of BC.

### 3.6 Univariate and multivariate Cox regression analysis of BC recurrence risk factors

Univariate and multivariate Cox regression models identified age, TNM stage, pathological grading, and increased urinary exosomal miR‐21 levels as risk factors for BC recurrence. Notably, multivariate Cox regression analysis confirmed that elevated urinary exosomal miR‐21 levels were an independent risk factor for BC recurrence (Table 5).

**TABLE 5:** Univariate and multivariate Cox regression analysis of risk factors for recurrence in patients with BC.

Variable	Univariate			Multivariate		
	HR	95% CI	*p* value	HR	95% CI	*p* value
Age	1.040	1.009–1.071	0.011	1.035	1.000–1.072	0.050
Gender	1.071	0.603–1.901	0.815			
TNM stage	14.325	5.642–36.371	<0.0001	10.189	3.772–27.523	<0.001
Grade	2.577	1.339–4.959	0.005	2.555	1.222–5.344	0.013
miR‐146a‐5p	0.019	0.001–0.683	0.030	0.352	0.009–14.380	0.581
miR‐93‐5p	0.384	0.054–2.717	0.337			
miR‐663b	7.188	0.491–105.221	0.150			
miR‐21	7.028	3.861–12.793	<0.0001	2.633	1.252–5.535	0.011
miR‐4454	1.187	0.538–2.622	0.671			
Urine cytology	0.739	0.265–2.058	0.563			

Abbreviation: HR, hazard ratio.

### 3.7 Prognostic value of urinary exosomal miR‐21 in BC patients

**FIGURE 6:**
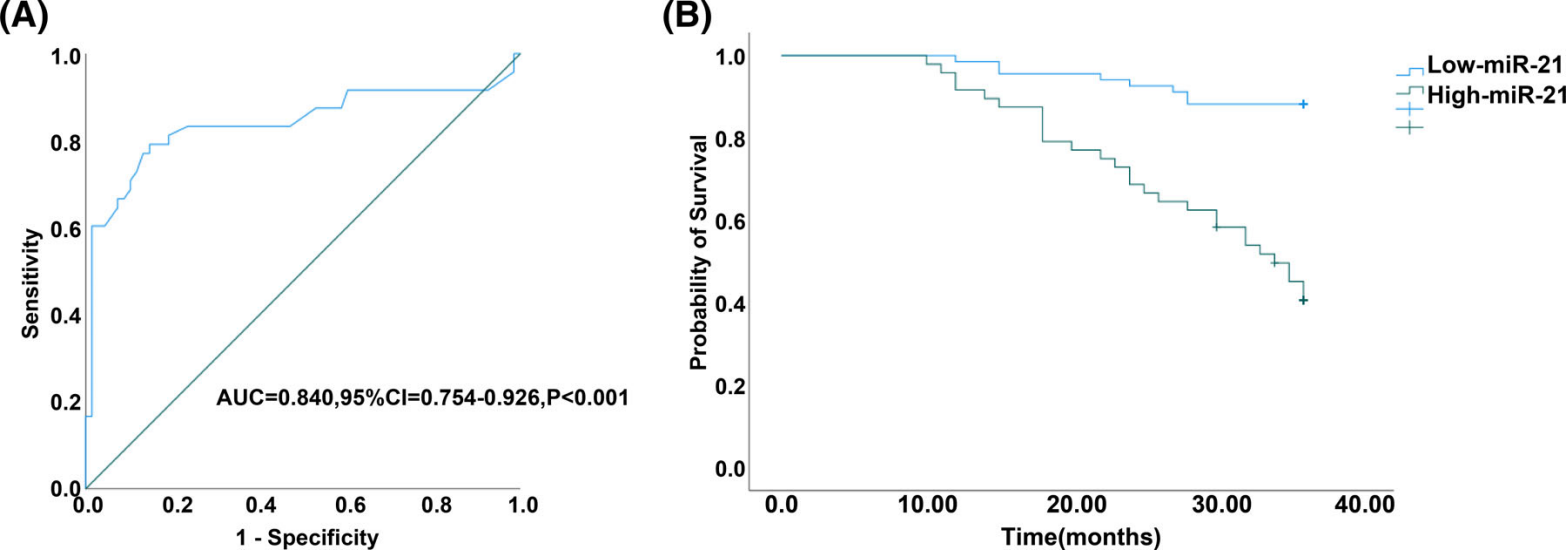
Relationship between miR‐21 and prognosis of patients with BC. Recurrence or death considered as a poor prognosis. ROC curve analysis was used to analyze the predictive value of urinary exosomal miR‐21 for poor prognosis. Using cut‐off value urinary exosomal miR‐21 as a threshold, BC patients divided into low‐miR‐21 and high‐miR‐21 groups, and 3‐year overall survival rates of BC patients in the two groups analyzed by Kaplan–Meier survival curves.

Patients were categorized based on urinary exosomal miR‐21 levels, with recurrence or death considered as a poor prognosis. Using a ROC curve (Figure 6A), a cut‐off value of 0.37 for miR‐21 was established. Patients with High‐miR‐21 (>0.37) exhibited significantly higher recurrence rate (79.17% vs. 14.71%) and mortality rates (58.33% vs. 11.76%) compared to those with Low‐miR‐21 group (Table 6, all *p* < 0.001). Kaplan–Meier survival curves revealed a lower 3‐year overall survival rate in the High‐miR‐21 group compared to the Low‐miR‐21 group (41.67% vs. 88.24%) (Figure 6B, *p* < 0.001), indicating that high urinary exosomal miR‐21 levels were indicative of poor prognosis in BC patients.

**TABLE 6:** Statistical analysis of recurrence and mortality rates in patients with BC.

	Recurrence	Mortality
Low‐miR‐21 group	10/68 (14.71%)	8/68 (11.76%)
High‐miR‐21 group	38/48 (79.17%)	28/48 (58.33%)
*p* value	<0.0001	<0.0001

*Note*: Using the cut‐off value urinary exosomal miR‐21 as the dividing line, BC patients were divided into the low‐miR‐21 and high‐miR‐21 groups. Enumeration data were expressed as number of cases (*n*) and percentages (%), and comparisons between the two groups were made using the χ^2^ test.

## 4 DISCUSSION

BC represents a complex disease characterized by diverse clinical, histological, and molecular profiles, and elucidating the intricate mechanisms underlying cell invasion and interactions within BC cells remains a challenge.^20^ Despite the gold standard diagnostic procedures for BC involving a combination of symptom assessment, medical history, and various laboratory tests including cytology, cystoscopy, imaging studies, and biomarker evaluation, these methods have limitations.^21^ Cystoscopy, for instance, is invasive, costly, and discomforting for patients, often yielding inconclusive results with false positives or negatives.^22^ In this context, there is a growing interest in exploring miRNAs as potential liquid biopsy biomarkers for BC, yet none have been widely adopted in clinical practice despite extensive research into urinary biomarkers for early, non‐invasive BC detection.^23^ Our study aimed to explore the diagnostic and prognostic potential of urinary exosomal miR‐21, revealing its promising diagnostic value and association with poor prognosis.

Advancements in laboratory technologies and our understanding of urine‐based BC biomarkers have propelled the development of non‐invasive liquid‐based biomarkers to complement urine cytology.^24^ Urinary biomarkers, including DNA, proteins, metabolites, various RNA types, and single nucleotide polymorphisms, have emerged as promising candidates for BC detection.^24^ Notably, miRNAs encapsulated within urine exosomes have garnered attention as potential diagnostic parameters for BC. Previous studies have identified several upregulated miRNAs in urinary exosomes of BC patients, including miR‐21, miR‐200c‐3p, miR‐205‐5p, miR‐4454, and miR‐720.^13^ In our investigation, we identified five miRNAs closely associated with BC progression, including miR‐146a‐5p,^10^ miR‐93‐5p,^11^ miR‐663b,^12^ miR‐21, and miR‐4454.^13^ These miRNAs, found to be highly expressed in BC tissues, participate in intercellular communication within the tumor microenvironment via the exosomal pathway. Consistent with previous findings, we observed significantly elevated levels of these miRNAs in urinary exosomes of BC patients compared to controls, with miR‐21 and miR‐4454 exhibiting the most pronounced differences in expression levels.

A plethora of circulating miRNAs has been identified in urine, holding promise as biomarkers for diagnosing BC.^25^ Leveraging urine‐derived miRNAs could offer a non‐invasive, targeted approach to BC diagnosis, presenting a favorable alternative to current invasive methods.^26^ In our study, we unveiled that urinary exosomal miR‐21 exhibited high diagnostic value for BC across both Ta‐T1 and T2‐T4 stages, underscoring its consistency as a diagnostic marker throughout different stages of BC. Notably, we assessed the diagnostic potential of single miRNAs as well as combined miRNA panels. Our analysis demonstrated that urinary exosomal miR‐21, along with the miRNA combined diagnostic panel Emdp‐miR and miR‐4454, displayed substantial diagnostic value for BC. Consistent with our findings, previous studies in other cancer types, such as triple‐negative breast cancer,^27^ have reported enhanced diagnostic efficacy with combined miRNA panels compared to individual miRNAs. Similarly, diagnostic panels incorporating multiple miRNAs have been shown to offer superior precision in diagnosing pancreatic ductal adenocarcinoma compared to relying on individual circulating miRNAs.^28^

Subsequently, we explored the correlation between clinical indicators such as age, sex, TNM stage, pathological grading of BC patients, and urinary exosomal miR‐21. Our analysis revealed that urinary exosomal miR‐21 levels were associated with TNM staging and pathological grading of BC. Furthermore, our experiments unveiled that urinary exosomal miR‐21, along with age, TNM stage, and pathological grading, served as independent risk factors for BC recurrence. Patients with high urinary exosomal miR‐21 levels exhibited higher recurrence and mortality rates, indicating a poorer prognosis. We corroborated our findings by examining large databases, which revealed high expression of exosomal miR‐21 in head and neck cancer and colorectal cancer (Figure S2). Additionally, existing literature consistently reports that elevated miR‐21 expression in BC patients has been linked to recurrence and invasiveness in BC, with studies demonstrating its enrichment in BC cell‐derived exosomes, facilitating BC cell migration and invasion.^29^ Moreover, altered expression of miR‐21‐5p in urinary exosomes has been implicated in renal dysfunction and renal fibrosis development, further emphasizing its diagnostic value in BC and urinary system‐related disorders.^30^

In this study, we highlight, for the first time, the high diagnostic value of miR‐21 and miR‐4454 alone for BC. Our findings reveal that urinary exosome miR‐21 exhibits robust diagnostic value for BC across both Ta‐T1 and T2‐T4 stages. Moreover, miR‐21, in conjunction with age, BC TNM stage, and BC grade, emerges as an independent risk factor for BC recurrence. Notably, BC patients with elevated levels of urinary miR‐21 are associated with a poor prognosis. This study introduces a novel urine biomarker with promising clinical application potential for the early diagnosis and prognosis of BC.

Furthermore, through the integration of sequencing data and comprehensive analysis of clinical samples, we established a diagnostic panel model, Emdp‐miR, comprising a combination of urinary exosomal miRNAs (miR‐146a‐5p, miR‐93‐5p, miR‐663b, miR‐21, and miR‐4454). The Logit panel model holds the potential to address limitations encountered in predicting ex‐ante events, offering valuable clinical insights for evaluation, diagnosis, and prediction.

However, it's essential to acknowledge several limitations of this study. The sample size was relatively small, based on the estimated minimum sample size, warranting further investigation with larger cohorts to validate our findings robustly. Additionally, during candidate miRNA selection, we relied solely on previous reports without conducting high‐throughput analysis of differential miRNAs in the urine of BC patients and healthy subjects. This approach might have overlooked some potential miRNAs relevant to BC diagnosis and prognosis. Furthermore, the relatively short follow‐up time limits the comprehensive assessment of long‐term outcomes. Future research directions will focus on addressing these limitations, aiming to enhance the understanding and clinical utility of urinary exosomal miRNAs in BC diagnosis and management.

## CONFLICT OF INTEREST STATEMENT

The authors declare no conflict of interest.

## Supporting information


**Figure S1.** Expression of miRNA‐21 under different internal reference gene controls.

**Figure S2.** Supporting Information.
