# Supplementary figures and images for: The value of urinary exosomal microRNA‐21 in the early diagnosis and prognosis of bladder cancer
Source: Kaohsiung J Med Sci. 2024 May 27;40(7):660–70. doi: 10.1002/kjm2.12845 (PMC11895653; doi:10.1002/kjm2.12845)

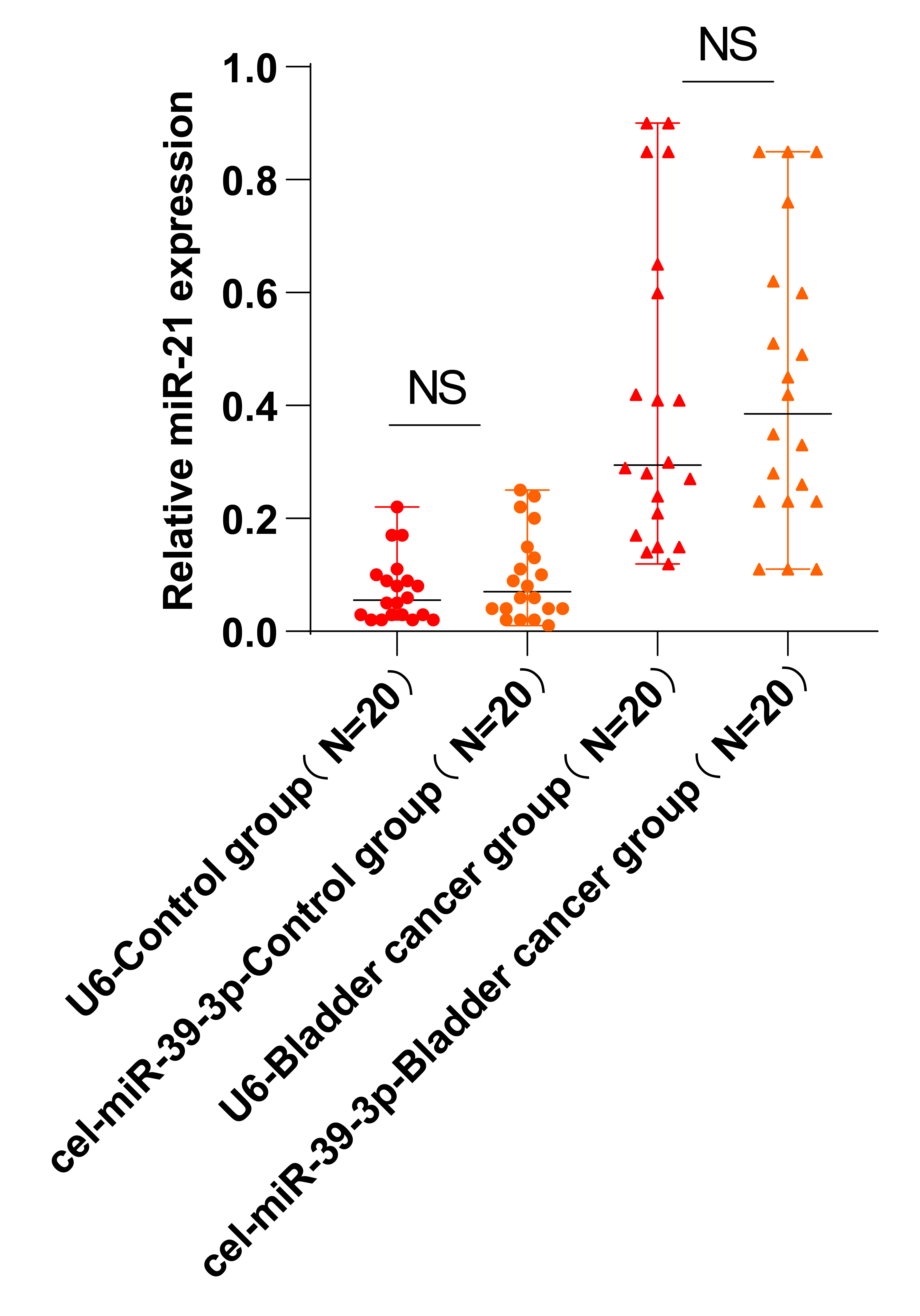

Supplement: Supplementary file 1 — Figure S1. Expression of miRNA‐21 under different internal reference gene controls. [file KJM2-40-660-s001.tiff]

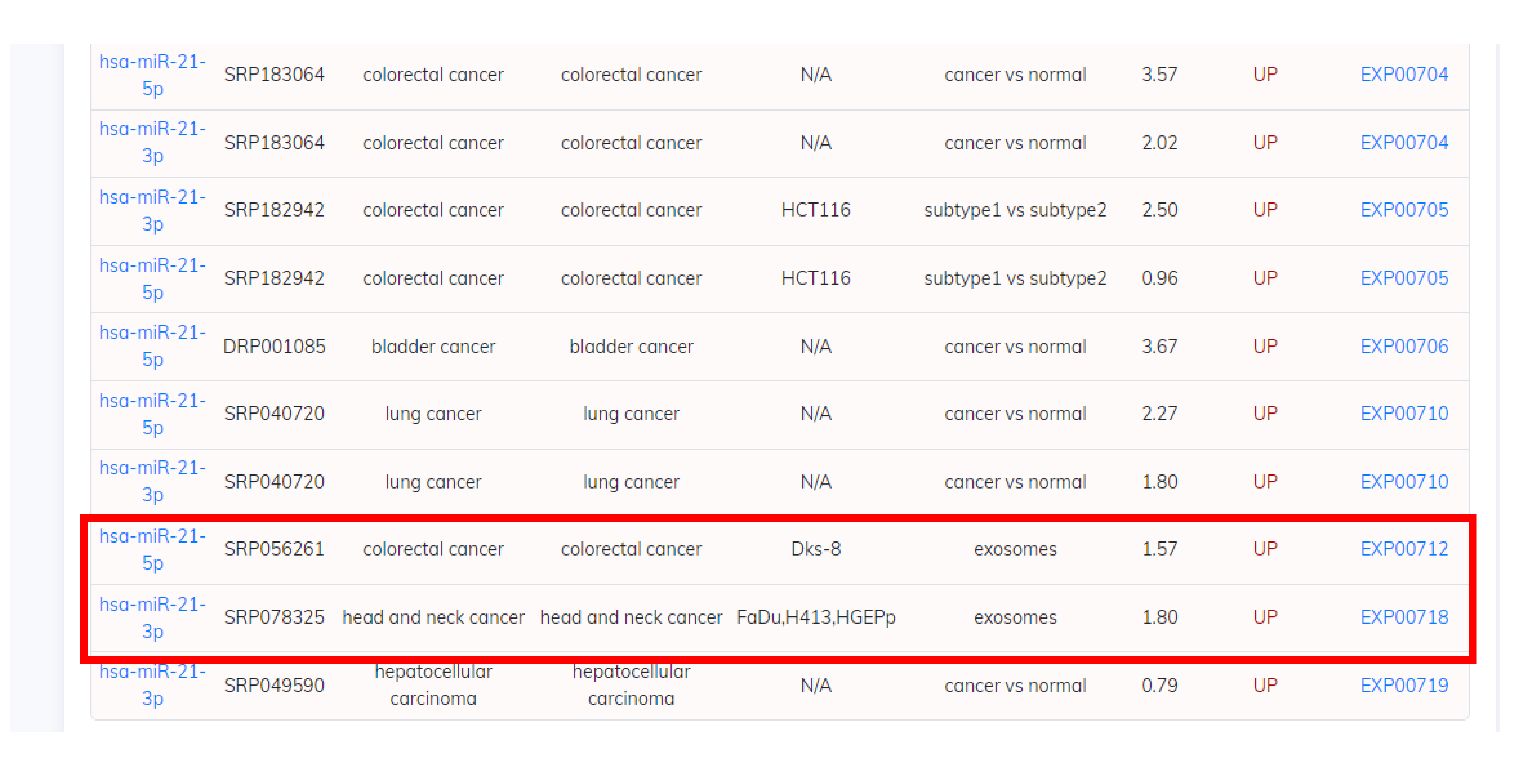

Supplement: Supplementary file 2 — Figure S2. Supporting Information. [file KJM2-40-660-s002.tiff]
